# Impacts of large-scale climatic disturbances on the terrestrial carbon cycle

**DOI:** 10.1186/1750-0680-1-7

**Published:** 2006-07-27

**Authors:** Tim Erbrecht, Wolfgang Lucht

**Affiliations:** 1 Potsdam Institute for Climate Impact Research, PO Box 6012303, D-14412 Potsdam, Germany; 2 Institute of Geoecology, Potsdam University, PO Box 601553, D-14415 Potsdam, Germany

## Abstract

**Background:**

The amount of carbon dioxide in the atmosphere steadily increases as a consequence of anthropogenic emissions but with large interannual variability caused by the terrestrial biosphere. These variations in the CO_2 _growth rate are caused by large-scale climate anomalies but the relative contributions of vegetation growth and soil decomposition is uncertain. We use a biogeochemical model of the terrestrial biosphere to differentiate the effects of temperature and precipitation on net primary production (NPP) and heterotrophic respiration (Rh) during the two largest anomalies in atmospheric CO_2 _increase during the last 25 years. One of these, the smallest atmospheric year-to-year increase (largest land carbon uptake) in that period, was caused by global cooling in 1992/93 after the Pinatubo volcanic eruption. The other, the largest atmospheric increase on record (largest land carbon release), was caused by the strong El Niño event of 1997/98.

**Results:**

We find that the LPJ model correctly simulates the magnitude of terrestrial modulation of atmospheric carbon anomalies for these two extreme disturbances. The response of soil respiration to changes in temperature and precipitation explains most of the modelled anomalous CO_2 _flux.

**Conclusion:**

Observed and modelled NEE anomalies are in good agreement, therefore we suggest that the temporal variability of heterotrophic respiration produced by our model is reasonably realistic. We therefore conclude that during the last 25 years the two largest disturbances of the global carbon cycle were strongly controlled by soil processes rather then the response of vegetation to these large-scale climatic events.

## Background

Anthropogenic emissions continuously add about 7000–8000 million metric tons of carbon to the atmosphere per annum [1]. Atmospheric carbon dioxide measurements show that the rate of increase of atmospheric CO_2 _varies substantially from year to year [2]. It is widely accepted that these variations are caused by the terrestrial biosphere through the processes of carbon uptake during photosynthesis and carbon release during soil respiration [3]. Additionally, strong disturbances such as large-scale fires can significantly alter the exchange of carbon between terrestrial ecosystems and the atmosphere. For example, up to 65 % of the observed CO_2 _growth rate in 1998 was attributed to burnt biomass in tropical and boreal regions [4]. In comparison, variations in the oceans [5], deforestation, and land use change are much smaller [6].

Uncertainty remains, however, regarding the relative influence of the driving climatic anomalies (temperature and precipitation anomalies) on the most prominent terrestrial carbon processes, namely vegetation growth (NPP) and soil decomposition (Rh). Numerical models of the land carbon cycle allow investigations of these relationships.

The two largest anomalies of atmospheric CO_2 _growth rate during the last 25 years are related to two large climatic disturbances – the increased planetary albedo after the eruption of Mount Pinatubo in 1991 and the strong El Niño event of 1997/98. The Pinatubo eruption was an extraordinary event because of the large amount of aerosols that were injected into the lower stratosphere where they were distributed around the globe, leading to a world-wide cooling of about 0.5°C [7]. The 1997/98 El Niño event was unusual in that it was extremely strong [8].

We use the LPJ model of terrestrial carbon and water cycles [9,10] to explore the covariability of climatic forcings and physiological responses (namely_NPP and Rh) of the terrestrial biosphere on a global scale as well as for selected latitudinal regions. Results show that a large fraction of the observed CO_2 _growth rate variability is controlled by varying soil organic matter decomposition rather than changing plant productivity. This sheds additional light on previous studies that highlighted connections between the interannual variability of atmospheric CO_2 _growth and net primary productivity [11-13].

## Results

### The 1992/93 sink event

**Figure 1:**
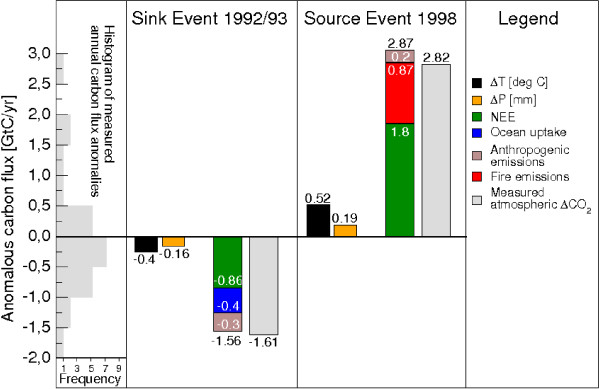
**Comparison of modelled and observed carbon flux anomalies**. Global carbon balance during 1992/93 and 1998 using modelled and measured data of carbon sources and sinks and atmospheric reference data. Negative fluxes denote carbon uptake. Negative values of measured atmospheric ΔCO_2 _denote a reduction it's rate of increase. Temperature and precipitation anomalies are calculated from the climate data set [29]. The histogram on the left is computed from measured carbon flux anomalies of last 25 years to illustrate the exceptional perturbation of the global carbon cycle in the two periods under investigation [1].

**Figure 2:**
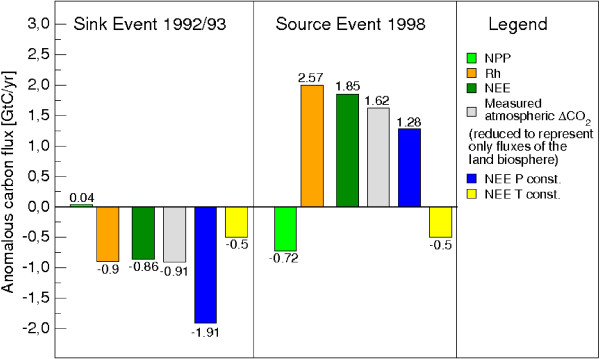
**Comparison of modelled and observed carbon flux anomalies**. Global modelling results (NPP, Rh, NEE) and atmospheric reference data representing changes in ecosystem physiology. Negative values of NEE, NPP and Rh denote carbon uptake by the land biosphere. Negative values of measured atmospheric ΔCO_2 _denote a reduction it's rate of increase.

In order to calculate the share of observed CO_2 _variability controlled by terrestrial ecosystem physiology we used state-of-the-art estimates of carbon flux anomalies from oceans, land-use change, anthropogenic emissions, and fires and reduced the measured atmospheric CO_2 _growth rate anomalies accordingly (see figure 1). An amount of -0.91 GtC/yr of the observed anomaly of -1.61 GtC/yr is found to have been caused by changes in NPP and Rh. The LPJ model computes -0.86 GtC/yr (fig. 2): it is in quantitative agreement with the observations. Higher than normal oceanic carbon uptake and reduced anthropogenic emissions contributed to the anomalous carbon flux in addition to vegetation productivity and soil respiration (fig. 1). There are no indications that wild fires contributed significantly to the observed post-Pinatubo flux anomalies [14].

What was the cause for this large anomalous sink – changes in NPP or Rh? The model shows that the pronounced reduction of atmospheric CO_2 _increase results from reduced soil respiration whereas NPP did not change (cf. [15-17]).

### The 1998 source event

The physiological response of the land biosphere during the strong 1997/98 El Niño event was diametrically opposite to that of the 1992/93 period. A large anomalous flux of 2.82 GtC into the atmosphere was measured in 1998 of which 1.62 GtC can be assigned to vegetation growth and soil decomposition processes of the terrestrial biosphere (fig. 1). LPJ simulates a value of 1.85 GtC due to stronger soil respiration that was partly counteracted by increased photosynthetic activity (fig. 2). The remaining 1.2 GtC of the anomalous land source consists of a small contribution from anthropogenic emissions (about 0.2 GtC) while the rest is attributable to carbon emissions from extensive fires in tropical, subtropical and boreal regions [4,18](fig. 1).

## Discussion

### The 1992/93 sink event

The quality of modelled soil carbon decomposition is less certain than that of NPP. Studies show, however, that the temporal variability of modelled vegetation activity is in good agreement with independent satellite data [17,19], indicating that the temporal variations of the associated simulated NPP are likely reliable. Because measured and modelled NEE, i.e. the difference of Rh and NPP, are found to be very similar in magnitude, we suggest that our simulation of the magnitude of anomalous soil respiration is plausible.

**Figure 3:**
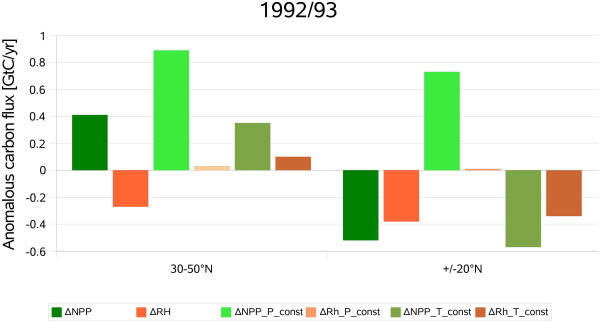
**Model results 1992/93**. Simulated anomalies of NPP and Rh in 1992/93 for temperate and tropical latitudes. P_const represents the simulations where the precipitation anomalies were removed. T_const represents the simulations where the temperature anomalies were removed. Positive values of NPP denote carbon uptake by vegetation and vice versa. Positive values of Rh denote carbon release from soils and vice versa.

While the results show that soil processes controlled the increased land sink it remains unclear why NPP did not react to the climatic anomalies and what the relative importance of changes in temperature and precipitation were. We carried out a factorial experiment where either post-Pinatubo temperature or precipitation anomalies (that is, only the 1992–94 anomalies) were removed in the simulations, i.e. monthly values of temperature and precipitation were replaced by their corresponding 1979–2003 averages. These simulations help to clarify how relevant the changes in temperature and precipitation were for the carbon budgets of of vegetation and soils. We find that if the global cooling after the Pinatubo eruption is removed (NEE T const.) the terrestrial carbon sink declines together with Rh, and higher values of NPP are achieved (fig. 3). When the observed precipitation anomalies, associated with a weak El Niño event that occurred during the same time period, are eliminated, negative temperature anomalies alone trigger a much larger sink than observed (fig. 3, P const). The increased net flux of carbon from the atmosphere into the biosphere results from enhanced NPP whereas Rh remains unchanged. In summary, it appears that the anomalously large sink post-Pinatubo sink was caused mainly by the dampening effects of lower temperatures on soil microbial activity (fig. 2), but that post-Pinatubo water-limitation of NPP weakened the strength of the sink.

### The 1998 source event

**Figure 4:**
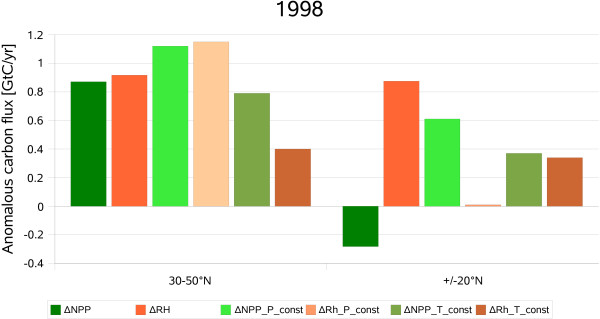
**Model results for 1998**. Simulated anomalies of NPP and Rh in 1998 for temperate and tropical latitudes. P_const represents the simulations where the precipitation anomalies were removed. T_const represents the simulations where the temperature anomalies were removed. Positive values of NPP denote carbon uptake by vegetation and vice versa. Positive values of Rh denote carbon release from soils and vice versa.

Warmer and wetter than usual conditions during 1998 stimulated both NPP and Rh in the wake of the large El Niño, with a particularly pronounced effect on soil respiration (fig. 2). The small anomalous increase in precipitation in the climate data deviates from the general assumption that El Niño episodes in the Amazon basin are characterized by extended dry seasons, lower wet season precipitation and shifts in spatial rainfall distribution [3]. The strong sensitivity of heterotrophic respiration to soil moisture content has also been observed in eddy covariance measurements [20]. Due to teleconnections El Niño periods also affect climatic conditions in the extratropics. For example, vegetation activity in the northern temperate regions is positively correlated with ENSO during the northern summer [21]. A pattern of negative NPP anomalies in the tropics and positive anomalies in the temperate zones is also produced by the LPJ model. However, the simulated global carbon balance is mainly determined by the differential response of tropical NPP and Rh. In the northern mid-latitudes, higher CO_2 _uptake by vegetation is counterbalanced by increased respiration due to higher temperatures (fig. 4). The dominant contribution of tropical ecosystems to the large land-atmosphere flux during El Niño conditions due to the opposite variation of NPP and Rh has been noted in other modelling studies as well [13,22].

Removing precipitation anomalies from the climate data results in a small terrestrial carbon sink that is triggered by increasing NPP in the tropics while reducing the carbon flux from soils (fig. 4, P const). The influence of the temperature anomalies is more important outside the low latitudes. Eliminating the positive temperature anomaly of 1998 reduces Rh significantly in the mid-latitudes whereas the carbon fluxes in and out of tropical ecosystems become balanced (fig. 4, T const). Thus the 1998 carbon source resulted from two mechanisms, a limitation of vegetation activity and a stimulation of soil decomposition in the tropics as well as temperature-driven acceleration (possibly via teleconnections) of heterotrophic respiration relative to NPP in northern temperate regions.

## Conclusion

We conclude that the two largest variations of the global carbon cycle observed during the last 25 years were predominantly controlled by soil processes rather than by vegetation activity. This adds a different perspective to previous analysis [11-13] that concentrated on contributions of global NPP anomalies to atmospheric CO_2 _growth rate anomalies. While NPP indeed plays an important role, analysis shows that in some periods changes in NPP alone do not explain the observed excursions of atmospheric carbon dioxide accumulation. This applies particularly to the two large events analysed in this paper, and in both cases changes in soil respiration explain the observed variability. Considering only the relationship between climate and NPP underestimates the true variability of the global carbon cycle. Vegetation growth and soil decomposition react differentially to anomalies in temperature and precipitation.

Observed and modelled NEE anomalies agree surprisingly well, suggesting that the LPJ model simulates the temporal variability of soil organic matter decomposition sufficiently. The model can hence be applied to the analysis of the land biosphere's modulation of atmospheric CO_2 _concentration and the biogeochemical effects of large-scale climate variations.

There are several implication for policy. First, long-term climate protection strategies aimed at full accounting of terrestrial carbon sources and sinks should focus on soil and vegetation processes equally. Currently, much more is known about vegetation responses to climate change than about soil processes [23]. Second, the terrestrial carbon cycle varies strongly in space and time. A monitoring regime therefore should take into account the characteristics of the dynamics observed and modelled for different global regions and temporal periods. Climatic events may strongly alter the short-term balance, rendering them untypical of the average behaviour.

## Methods

### The LPJ DGVM

The LPJ Dynamic Global Vegetation Model [9,10] is a biogeochemical model of fluxes of carbon and water in terrestrial vegetation and soils. Carbon uptake during photosynthesis is estimated using the Farquhar-Collatz scheme which is coupled to two soil layers [24,25]. Assimilated carbon is allocated to four pools (leaves, sapwood, heartwood and fine roots) following allometric and functional relationships [9]. Carbon from dead biomass enters above- and belowground litter pools and is then transferred to a fast and slow decomposing soil carbon pool. Soil organic matter decomposition is calculated using a modified Arrhenius formulation [26] which implies a decline in apparent Q_10 _with temperature, as well as an empirical soil moisture relationship [27].

We performed simulations with 0.5 degrees spatial resolution (59199 grid cells) using an interpolated climatology of monthly values of temperature, precipitation and radiation [28,29]. A land cover data set produced at the University of Maryland provided a realistic distribution of global crop lands [30]. The LPJ-DGVM has been extensively validated using various data from atmospheric measurements, active and passive remote sensing data, and flux measurements. It was shown that the model is capable of simulating large-scale structure, distribution and phenology of global vegetation [9,17] as well as the inferred seasonal cycles of soil moisture [31], evapotranspiration and runoff [10].

## Competing interests

The author(s) declare that they have no competing interests.

## Authors' contributions

Tim Erbrecht carried out the LPJ simulations and participated in the analysis and interpretation of modeling results, and wrote the manuscript. Wolfgang Lucht participated in data analysis and interpretation.
